# A Novel Method for the Determination of Vancomycin in Serum by High-Performance Liquid Chromatography-Tandem Mass Spectrometry and Its Application in Patients with Diabetic Foot Infections

**DOI:** 10.3390/molecules23112939

**Published:** 2018-11-10

**Authors:** Min Liu, Zhi-Hui Yang, Guo-Hui Li

**Affiliations:** 1 Department of Pharmacy, National Cancer Center/National Clinical Research Center for Cancer/Chinese Academy of Medical Sciences and Peking Union Medical College, Beijing 100021, China; 2 Institute of Aviation Medicine of Air Force, Beijing 100142, China; zhyanghos@163.com

**Keywords:** vancomycin, norvancomycin, high performance liquid chromatography—tandem mass spectrometry, Diabetic Foot Infection (DFI), MRSA, Serum

## Abstract

A novel, precise, and accurate high-performance liquid chromatography-tandem mass spectrometry (Q-trap-MS) method was developed, optimized, and validated for determination of vancomycin in human serum using norvancomycin as an internal standard. Effect of different parameters on the analysis was evaluated. ZORBAX SB-C_18_ column (150 × 4.6 mm, 5 μm) using water (containing 0.1% formic acid, *v*/*v*)–acetonitrile (containing 0.1% formic acid, *v*/*v*) as a mobile phase was chosen. The calibration curve was linear over the concentration ranges of 1 to 2000 ng/mL for vancomycin. The limit of detection (LOD) and limit of quantification (LOQ) for vancomycin were 0.3 and 1.0 ng/mL. Recoveries were between 87.2 and 102.3%, which gave satisfactory precision. A total of 100 serum samples (from 50 patients with diabetic foot proven Gram-positive infection and 50 nondiabetic patients with pneumonia requiring hospitalization and antibiotic therapy) were analyzed by this method. The trough vancomycin concentrations of diabetic foot infection (DFI) patients and nondiabetic patients were 8.20 ± 2.83 μg/mL (range: 4.80–14.2 μg/mL) and 15.80 ± 5.43 μg/mL (range: 8.60–19.5 μg/mL), respectively. The method is sensitive, precise, and reproducible, it could be applied for routine laboratory analysis of vancomycin in serum samples.

## 1. Introduction

**Figure 1:**
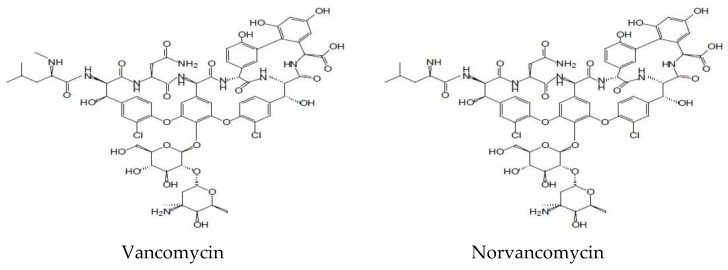
Structure of vancomycin and norvancomycin.

Diabetic foot infection (DFI) is a serious complication of diabetes, which can be caused by a variety of microorganisms. Among the common pathogens, Gram-positive cocci, especially *Staphylococcus aureus*, have often been reported to be related to DFIs [1,2,3]. Methicillin-resistant *Staphylococcus aureus* (MRSA) often occurs when patients are hospitalized or treated in community hospitals, it can be detected in approximately 20% DFIs patients, which can prolong healing time and cause a lot of serious problems. This deadly pathogen is not conducive to wound healing and increases the risk of lower limb amputation. Antibiotic therapy is necessary when treating with aerobic Gram-positive cocci (such as MRSA) [4]. Vancomycin, (Figure 1), a kind of glycopeptide antibiotic, is often recommended for severe skin/soft tissue infections caused by MRSA, such as DFIs and so on [5,6]. Hypoxia and ischemia of lower limbs occurs in most DFIs patients, which can reduce the tissue penetration of antibacterial agents and may significantly change the pharmacokinetics of antibiotics in serum and tissues [7]. Previous studies have revealed that vancomycin can penetrate into the interstitial fluid [8,9,10]. Although the vancomycin serum concentrations of these patients are similar, the level of tissue exposure varies greatly. Skhirtladze et al. [8] once compared skin penetration of vancomycin in different patients (with or without diabetes), the results showed that the plasma concentrations of vancomycin in diabetic patients and nondiabetic patients were similar, anyway, in this study, only six diabetic patients were included and there was no study showing that whether there was a difference of blood concentration of vancomycin between DFI patients and patients without DFI.

A lot of methods have been established for the determination of vancomycin, such as HPLC [11,12,13], capillary electrophoresis [14,15], and so on [16,17,18]. HPLC is a proper method due to its low cost and wide range of application. However, analysis performed on conventional HPLC lacks of sensitivity [11,12,13]. Capillary electrophoresis is also commonly used in determination of vancomycin, the advantages of the method includes low sample consumption, high separation efficiency, short analysis time, and so on, but the reproducibility of this method is poor [14,15]. Up to now, HPLC-MS/MS methods are the most common used methods for determination of vancomycin [19,20,21,22]. The methods were successfully applied into quantify vancomycin in different matrices, but each of them had its limitations. The methods either required a long analysis time [19], lacked of internal standard [19], or had insufficient sensitivity [20,21,22].

So far, reports of determination of vancomycin using Q-trap MS are seldom, Schmitt et al. [23] once established a Q-trap MS method for determination of vancomycin in rabbit serum, but they lacked studies of matrix effects and stability, which caused the incomplete of the method. In this research, a reliable method based on Q-trap-MS was developed to determine vancomycin; the methodological parameters were verified comprehensively. The proposed method is simple, precise and accurate; it is applicable to determination of vancomycin in multiple samples.

## 2. Material and Methods

### 2.1. Chemicals, Reagents and Samples

Vancomycin and norvancomycin were obtained from Ehrenstorfer (Augsburg, Germany, purity grade >95.0%). Methanol (MeOH, LC/MS grade), acetonitrile (MeCN, LC/MS grade), and formic acid (98%) were purchased from Sigma-Aldrich (St. Quentin Fallavier, France). Milli-Q-System (Millipore, Guyancourt, France) was adopted for purifying water. A nylon membrane filter (pore size: 0.22 μm) was obtained from Jinteng Laboratory Equipment Co., Ltd. (Tianjin, China). 

### 2.2. Apparatus

The HPLC-Q-trap-MS system consisted of an AB SCIEXQTRAP^®^ 6500 mass spectrometer (AB SCIEX LLC. Redwood City, CA, USA) and HPLC chromatographic analysis system (Spark Holland B.V., Holland) with Alias^TM^ Autosampler, SPH 1240 Gradient Pump.

### 2.3. Preparation of Quality Controls and Standard Solutions

The standard stock solutions of vancomycin and norvancomycin (IS) were prepared by dissolving accurately weighed standard compound in deionized water at 1.0 mg/mL. Then, the working solutions of vancomycin were diluted serially with deionized water to achieve final concentrations of 10, 20, 50, 100, 200, 500, 1000, 2000, 5000, 10,000, and 20,000 ng/mL. Ten microliters of the diluted solutions were added to blank serum to obtain final concentrations ranged from 1 to 2000 ng/mL.

The IS working solution was diluted with deionized water to give a final concentration of 100 ng/mL. Quality control (QC) samples were prepared in 100 μL of blank serum by adding 10 μL of serially diluted solutions of the substance to determine the limit of quantification, as well as 2 ng/mL (low), 20 ng/mL (medium), and 400 ng/mL (high) concentrations. The L, M, and H of QCs were analyzed at least in duplicate. All solutions were stored at −20 °C and brought to room temperature before use.

### 2.4. HPLC-Q-Trap-MS Conditions

The chromatographic separation was performed on an Agilent ZORBAX SB-C_18_ (4.6 × 150 mm, 5 μm). The mobile phase consisted of water (containing 0.1% formic acid, *v*/*v*) and acetonitrile (containing 0.1% formic acid, *v*/*v*) and the flow rate was set at 0.5 mL/min. An isocratic elution was performed with 30% of acetonitrile. The total run time was 8 min. The injection volume was 10 μL.

The mass spectrometer was operated in the positive ion electrospray mode with curtain gas flow rates of 20 psi. The ion spray voltage and the source temperature were set at 5000 V and 300 °C, respectively. Multiple reaction monitoring (MRM) was adopt for data acquisition. The optimized precursor-to-product ion transitions monitored for vancomycin [M + 2H]^2+^ were *m*/*z* 725.8 → 144.2 with declustering potential (DP) 50 V, collision energy (CE) 20.4 V, and *m*/*z* 725.8 → 100.1 with declustering potential (DP) 50 V and collision energy (CE) 50.4 V, respectively. The optimized precursor-to-product ion transitions monitored for norvancomycin [M + H]^2+^ were *m*/*z* 718.8 → 144.3 with DP 40 V, CE 19 V and *m*/*z* 718.8 → 99.7 with DP 40 V and CE 65 V, respectively.

### 2.5. Sample Source and Pretreatment

Clinical samples were collected at Air Force General Hospital, PLA. Fifty patients with DFIs and 50 nondiabetic patients with pneumonia who had been treated with vancomycin for a suspected or proven Gram-positive infection and met the TDM guidelines for vancomycin were included. Human blank serum was obtained from healthy blood donors not using vancomycin (Air Force General Hospital, PLA.). The study was approved by the hospital ethics committee. Every patient was given a sufficient description of the study and signed an informed consent prior to the study.

The pretreatment followed for all samples was 10 μL of the IS was added to 20 μL of the standard and unknown serum samples and mixed vigorously for 20 s. Five-hundred microliters of acetonitrile was added, vortexed for 1 min, and centrifuged for 6 min at 12,000 rpm. After transferring the supernatant to a fresh centrifuge tube, it was evaporated to dryness under a stream of nitrogen at room temperature. Two-hundred microliters of mobile phase was added to the resulting residue and vortexed for 1 min; 100 μL was analyzed.

### 2.6. Method Validation

#### 2.6.1. Specificity

The specificity of the method was evaluated by comparing the chromatography of blank serum samples with that of blank serum spiked with vancomycin and/or norvancomycin (IS) and serum samples of patients treated with vancomycin.

#### 2.6.2. Linearity and Sensitivity

The calibration curve for vancomycin was validated with a series of standard samples in the range of 1 to 2000 ng/mL in serum. The linearity of the calibration curves was generated by plotting the ratio of the analyte to the IS signal versus the analyte concentration and fitted by a weighted linear least-squares linear regression with a weighting factor of 1/y, where y is the peak area ratio of vancomycin versus IS. The calibration curve was obtained by analyzing three replicates of the QC samples

Sensitivity of the method was evaluated in terms of LOQ which was determined based on two criteria as follows, (1) the analyte response at the LOQ be at least 10 times the response compared to the blank response and (2) the analyte peak must be identifiable, discrete, and reproducible with an accuracy (relative error) and precision (relative standard deviation, RSD) within 15%.

#### 2.6.3. Precision, Accuracy and Matrix Effect

The intraday accuracy and precision were obtained by analyzing seven replicates of the QC samples at three concentrations (400, 20, and 2 ng/mL) in a day, while the interday accuracy and precision were conducted by determining the replicates on three separate days. Freshly prepared calibration standards were used to measure the concentration of each QC sample. The relative standard deviation (RSD) of the replicates was used to represent the precision. The matrix effect was determined by dividing the slopes of calibration curves of vancomycin in blood matrix and mobile phase.

The accuracy was determined based on the following criteria. (1) The mean value should not exceed 15% of the nominal concentration and (2) for the LOQ, it should not exceed 20%. Similarity, the RSD of precision for each concentration level should not be deviated by more than ±15%, while for the LOQ, it should not exceed 20%.

#### 2.6.4. Recovery

The recovery of vancomycin was measured on QC samples at three concentrations (400, 20, and 2 ng/mL) in five replicates. The extraction recovery was assessed by comparing the peak area responses for the extracted samples of QC with those of the extracts of blank serum samples added with the same concentration of IS.

#### 2.6.5. Stability

In order to evaluate the stability of vancomycin in serum, four studies (bench-top stability, freeze-thaw stability, long-term stability, and autosampler stability) were employed. For the bench-top stability during handing, QC samples were prepared and kept room temperature (25 °C) for 6 h. Freeze (−80 °C)–thaw (room temperature) stability of the analytes was tested with three free-thaw cycles. For long-term stability, the QC samples were stored at −80 °C for 30 days. Stability of sample in autosampler was determined by analyzing the targets after kept in an autosampler at room temperature for 12 h.

## 3. Results

### 3.1. HPLC-MS Instrument Method Development

Q-Trap-MS is widely used due to its advantages high sensitivity, good repeatability, and wide dynamic range [24]. Vancomycin is a polar compound [25], so a positive ESI mode was employed.

**Figure 2:**
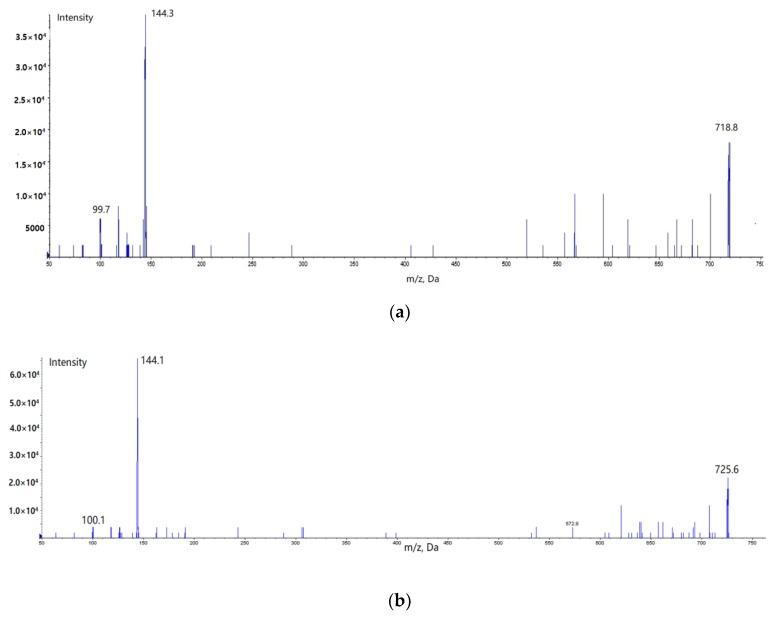
Product ion mass spectra of [M + H]^2+^ for (**a**) vancomycin (718.8 → 144.3, 99.7) and (**b**) norvancomycin (725.6 → 144.1, 100.1).

Step 1: The appropriate precursor ion to be fragmented should be confirmed first. Vancomycin can be easily protonated to form doubly-charged protonated molecular ions in the positive ESI mode since it possesses nitrogen-containing functional groups [13]. In the chosen scan mode, as seen in Figure 2, the target was identified according to its mass in the form of a doubly-charged ion.

Step 2: The product ions scan mode was used to select suitable fragment ions of the precursor ion. More than three fragment ions for the target were usually tested and among them, two more sensitive ions are chosen for the final mode (See in Figure 2).

Step 3: Both CE and DP should be optimized since are critical parameters which can affect the sensitivity of the target. The optimizing values were set from 0 to 180 eV during the optimizing process of CE and DP in MRM mode. The value of the highest abundance was chosen for the targets.

### 3.2. Sample Extraction Method Development

#### 3.2.1. Optimization of the Pretreatment Method

In this research, we investigated the matrix effect first, the matrix effect was examined by calculating the percentage (C%) of signal enhancement or suppression, according to Equation (1): C% = (1 − Ss/Sm) × 100, where Ss is the slope of matrix-matched calibration curve and Sm is the slope of standard solution calibration curve [26]. In our work, C% <±15% is acceptable. The values of C% for vancomycin and norvancomycin were −6.8% and 5.3%, respectively, which means no obvious matrix effect was observed. Thus, serum samples were directly extracted with acetonitrile.

#### 3.2.2. Effects of Different Conditions on the Recovery

Extraction solution and extraction time could greatly affect the extraction efficiency. To achieve the best extraction efficiency, these parameters were optimized. Results showed that acetonitrile has the highest recoveries (87.2–102.3%) compared to n-hexane, methanol, and ethy lacetate. The extraction time for the highest recovery in acetonitrile was 6 min at 25 °C.

### 3.3. Method Validation

The method validation was carried out in accordance with Food and Drug Administration (FDA) regulations for the validation of bioanalytical methods [27].

#### 3.3.1. Specificity

**Figure 3:**
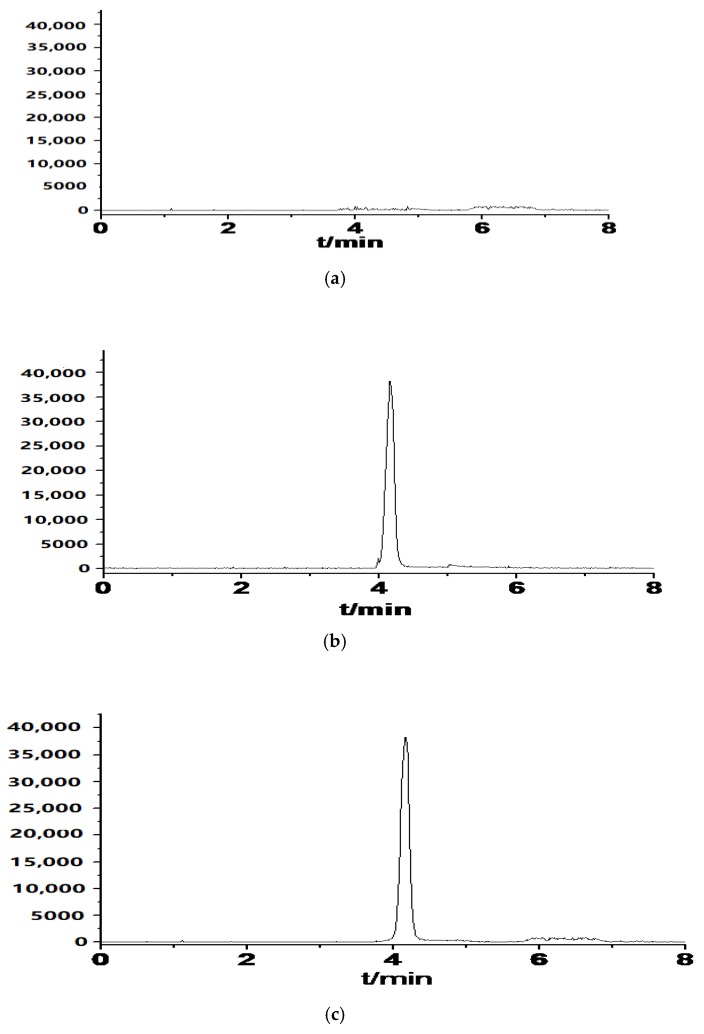

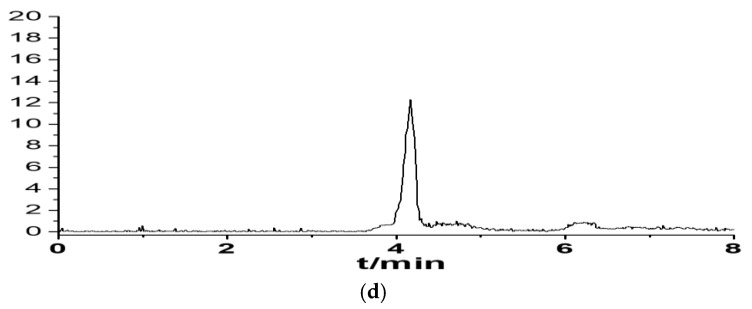
Typical chromatograms of (**a**) blank serum, (**b**) vancomycin standard in deionized water (500 ng/mL), (**c**) vancomycin standard in serum (patients after treated with vancomycin), (**d**) blank sample spiked with vancomycin (1 ng/mL).

The representative chromatogram of the blank serum (a), vancomycin standard in deionized water (b), vancomycin standard in serum (c) were shown in Figure 3. It can be seen that there was no significant interference with the vancomycin at its retention times in blank serum.

#### 3.3.2. Calibration and LOQ

Eleven different concentrations from 1 to 2000 ng/mL were analyzed for the calibration standards. The typical linear regression equation of the calibration curve was *y* = 597*x* + 5.6 × 10^3^ for vancomycin and it exhibited a good linearity (*R*^2^ = 0.9999). 

The LOQ of vancomycin was 1 ng/mL, which was significantly lower than the values reported in other methods [11,12,20,21,22], and it was significantly lower than the minimum effective concentration of vancomycin in serum.

#### 3.3.3. Precision and Accuracy

Table 1 showed the intra- and interday accuracy and precision using QC samples at 400, 20, and 2 ng/mL (representing high, medium, and low concentrations, respectively). It could be seen that the intra- and interday precision ranged from 3.21 to 6.67% and 4.11 to 7.14%, respectively, representing which was within the acceptance limit of 15%. 

#### 3.3.4. Extraction recovery

As shown in Table 2, the results indicated that the method showed high recovery in serum. The recoveries were over 85 %, indicating the suitability of the proposed method for the determination of vancomycin. The precision was satisfactory with a RSDs below 4% for spiked samples.

#### 3.3.5. Stability

Stability of vancomycin in serum was evaluated at three different concentrations. As shown in Table 3, vancomycin was stable in serum at 4 °C and 25 °C for 24 h, after three freezing and thawing cycles and stored at −80 °C for 30 days. 

### 3.4. Clinical Application

#### 3.4.1. Patients and Methods

Fifty diabetes mellitus type 2 patients with known or suspected Gram-positive DFI (Wagner score grade 3/4, equivalent to Texas classification of at least B3), and 50 nondiabetic patients with pneumonia requiring antibiotic therapy from 2014 to 2016 were included. A total of 100 trough serum samples of vancomycin were analyzed with this method. Steady-state serum vancomycin concentration for a dosing regimen of 1g q12h were compared between the two groups. Blood collection should be started around 48 h after the administration of vancomycin. The concentration of vancomycin should be maintained between 10 to 20 mg/L in adult patients based on a guideline of the therapeutic drug monitoring of vancomycin by Chinese Pharmacological Society [28]. The estimated Cl_Cr_ was calculated from serum creatinine using the Cockcroft-Gault formula [29]. CrCl = (140-age in years) × body weight in kg/(serum creatinine in µmol/mL × 815) × 0.85 for females). Statistical analysis was performed by SPSS 25.0.

#### 3.4.2. Results

Dose and dosage interval for all patients included in this experiment were the same (1g q12h). As listed in Table 4, there were more patients that achieved target trough concentration of vancomycin among nondiabetic group compared with DFI group (40(80.0%) vs. 18(36.0%), *p* = 0.020). The trough concentration of vancomycin in diabetic foot patients was significantly lower than that of nondiabetic patients (8.20 ± 2.83 μg/mL (range: 4.80–14.2 μg/mL) vs. 15.80 ± 5.43 μg/mL (range: 8.60–19.5 μg/mL), *p* = 0.004). CrCl in diabetic foot patients was higher than that in nondiabetic patients (126.14 ± 42.36 mL/min vs. 105.76 ± 38.66 mL/min, *p* = 0.005), which may be an important cause of the significant difference in serum concentration of vancomycin in the two groups.

## 4. Discussion

In recent days, a phenomenon which called ‘augmented renal clearance’ (ARC) was often mentioned by the researchers. ARC mainly referred to the enhancement of elimination of circulating solutes and exhibited as a hypermetabolic status. The presence of hypermetabolism in patients with ARC, which results in increased renal blood flow and the rate of glomerular filtration [30,31,32,33], may lead to the treatment failure of antibiotics [34,35,36,37]. Under normal conditions, target tissue and blood concentration can be obtained using the standard dosage of vancomycin, but in ARC patients, drug concentration in vivo was usually abnormal.

Our results showed that the trough concentration of DFI patients was significantly lower than that of patients without diabetes. Previous study showed that the plasma concentration of vancomycin in diabetic patients and nondiabetic patients were similar [8]. However, only six diabetic patients and six nondiabetic patients were enrolled, and there was no significantly difference in the average creatinine clearance of the two groups. But in our study, the Cl_Cr_ of DFI patients were significantly higher than that of nondiabetic foot infections, with a mean value of 126.14 mL/min.

Although the normal limits of Cl_Cr_ were defined as 130 mL/min/1.73 m^2^ for male and 120 mL/min/1.73 m^2^ for female, respectively [38], Cl_Cr_ values greater than 120–130 mL/min/1.73 m^2^ were defined as increased renal clearance, regardless of gender [38,39]. Another study recommended that Cl_Cr_ > 150 mL/min/1.73 m^2^ and >120 mL/min/1.73m^2^ were used for definition of hyperfiltration for young and elderly adults, respectively [40]. In the study, we chose Cl_Cr_ > 120 mL/min/1.73 m^2^ as the critical value of hyperfiltration state, considering that the patients in our study were mainly elderly, with an average age of 61 years. Diabetics in our study are all DFIs patients; most of them had poor glycemic control, complicated with diabetic nephropathy stage I, which appears glomerular hyperfiltration. It means that diabetic patients in our study have high level of Cl_Cr_, which can affect the clearance of hydrophilia antibiotics such as vancomycin.

Vancomycin is a typical hydrophilic antibiotic excreted mainly by kidney. Its clearance is closely related to Cl_Cr_, showing time-dependent activity [41,42,43,44]. We have known that the kidney could become enlarged and the glomerular filtration rate becomes abnormal in the early stages of diabetes [30]. Many studies have shown that the kidneys show a persistent increase in glomerular filtration rate in diabetic patients, ranged from 20 to 30%, and in some untreated diabetic patients may be as high as 30 to 40% [45]. A lot of studies have pointed out that primary abnormalities in vascular control can increase renal blood flow and vasodilate the renal vessels [36,37,38,39,40,41]. Gruden et al. [46] reported an improvement between 25 to 50% of GFR in more than 70% patients with type 1 diabetes at the early stages of diagnosis or in the early years of the disease and before the onset of proteinuria, while in type 2 diabetes mellitus patients, high glomerular filtration was found in 50% patients in the early years of the disease [47,48,49]. Therefore, the clearance of drugs excreted through the kidney, such as vancomycin, was elevated by the augmented glomerular filtration and the increased renal blood flow; it was the most likely risk factor for unsatisfactory vancomycin trough concentration. In some recent studies, the use of vancomycin loaded doses or continuous infusion may be strategies to avoid the phenomenon [41]. In the future, more DFI patients should be involved to be studied on vancomycin serum concentration compared with nondiabetics to insure whether high glomerular filtration is an important risk factor of failure of hydrophilic antibiotic on anti-infective therapy of DFIs.

## 5. Conclusions

In this study, a precise and sensitive quantitative method was developed and validated for analysis of vancomycin in serum by HPLC-Q-Trap-MS. The method exhibited excellent precision, recovery and curve linearity for the analyte. The method can be successfully applied to analysis of the target. The results indicated that HPLC-Q-Trap-MS could serve as a highly interesting analytical alternative for bioanalysis.

## Figures and Tables

**Table 1:** Intra and interday precision and accuracy of vancomycin in serum.

Concentration (ng/mL)	Intraday (*n* = 7)			Interday (*n* = 7)		
	Measured Conc (ng/mL)	Precision, RSD (%)	Accuracy (%)	Measured Conc (ng/mL)	Precision RSD (%)	Accuracy (%)
400	396.66 ± 22.17	3.21	99.17	394.38 ± 23.75	4.11	98.60
20	20.98 ± 1.52	5.17	104.90	21.14 ± 1.91	5.86	105.70
2	2.10 ± 0.19	6.67	105.00	1.99 ± 0.18	7.14	99.50

**Table 2:** Extraction recovery of vancomycin in serum.

Analyte	Concentration (ng/mL)	Extraction Recovery (%)	
		Mean ± SD	RSD
Vancomycin	400	99.72 ± 2.23	1.26
	20	94.94 ± 3.88	1.59
	2	92.51 ± 4.18	3.67

**Table 3:** Stability of vancomycin in serum under various storage conditions.

Storage Condition	Concentration (ng/L)	Mean ± SD (ng/L)	RSD %
Autosampler (4 °C) temperature for 24 h	2	2.20 ± 0.21	8.76
	20	21.67 ± 1.92	8.13
	400	408.16 ± 7.88	2.19
Room temperature (25 °C) for 24 h	2	2.24 ± 0.25	3.33
	20	22.14 ± 2.24	6.51
	400	406.12 ± 6.92	2.26
−80 °C for 30 days	2	2.29 ± 0.24	8.91
	20	21.01 ± 1.73	8.77
	400	420.11 ± 7.95	2.11
Freezing and thawing cycles	2	2.30 ± 0.21	8.12
	20	22.18 ± 1.77	6.51
	400	411.187 ± 9.91	1.52

**Table 4:** Comparison of DFI group and nondiabetic group.

	DFI Group (*n* = 50)	Nondiabetic Group (*n* = 50)	χ^2^/t	*p*
Samples of achieving target trough concentration	18(36.0%)	40(80.0%)	5.386	0.020
Trough Concentration of vancomycin (μg/mL)	8.20 ± 2.83	15.8 ± 5.43	3.781	0.004
Cl_Cr_ (mL/min)	126.14 ± 42.36	105.76 ± 38.66	2.891	0.005
